# Author Correction: Genetic dissection of clonal lineage relationships with hydroxytamoxifen liposomes

**DOI:** 10.1038/s41467-018-06927-2

**Published:** 2018-10-19

**Authors:** Ryan C. Ransom, Deshka S. Foster, Ankit Salhotra, Ruth Ellen Jones, Clement D. Marshall, Tripp Leavitt, Matthew P. Murphy, Alessandra L. Moore, Charles P. Blackshear, Elizabeth A. Brett, Derrick C. Wan, Michael T. Longaker

**Affiliations:** 1 Department of Surgery, Division of Plastic and Reconstructive Surgery, Stanford University School of Medicine, Stanford, 94305 CA USA; 2 Institute for Stem Cell Biology and Regenerative Medicine, Stanford University School of Medicine, Stanford, 94305 CA USA

Correction to: *Nature Communications* 10.1038/s41467-018-05436-6, published online 30 July 2018

In the original version of this article, the authors inadvertently omitted Elizabeth A. Brett, who contributed to the generation of the histology figures, from the author list. This has now been corrected in both the PDF and HTML versions of the Article.

