# Supplementary material for: Genetic dissection of clonal lineage relationships with hydroxytamoxifen liposomes
Source: Nat Commun. 2018 Jul 30;9:2971. doi: 10.1038/s41467-018-05436-6 (PMC6065311; doi:10.1038/s41467-018-05436-6)
Supplement: Supplementary file 1 — Supplementary Information [file 41467_2018_5436_MOESM1_ESM.pdf]

a

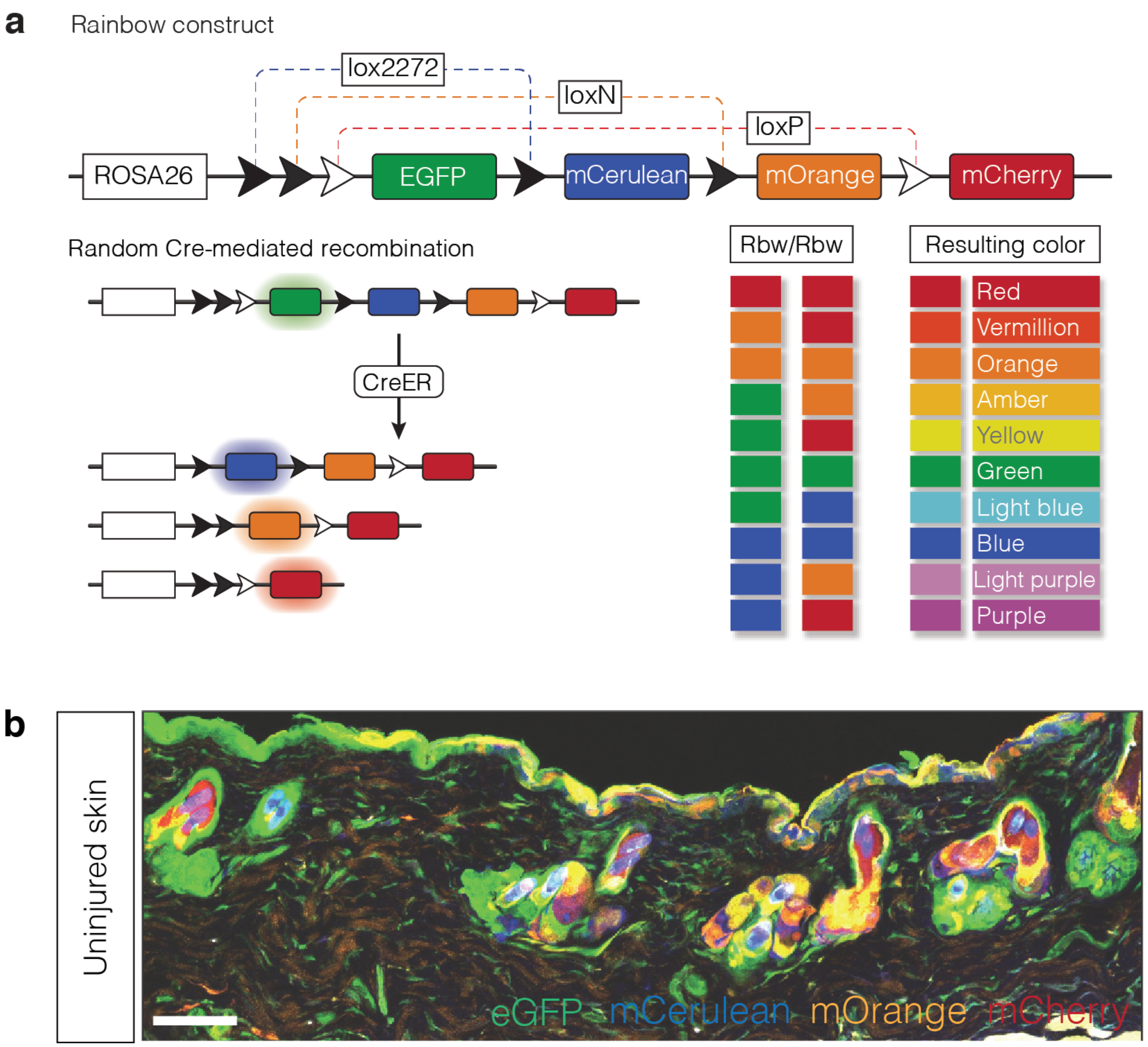

Supplementary Fig. 1. Application of LiTMX to uninjured skin in homozygous Rainbow mice

(**a**) Schematic of the homozygous Rainbow construct showing random Cre-mediated recombination and resultant cellular color code.

(**b**) Activation of the Rainbow construct 1 week after application of LiTMX to uninjured skin. Scale bars represents 200um.

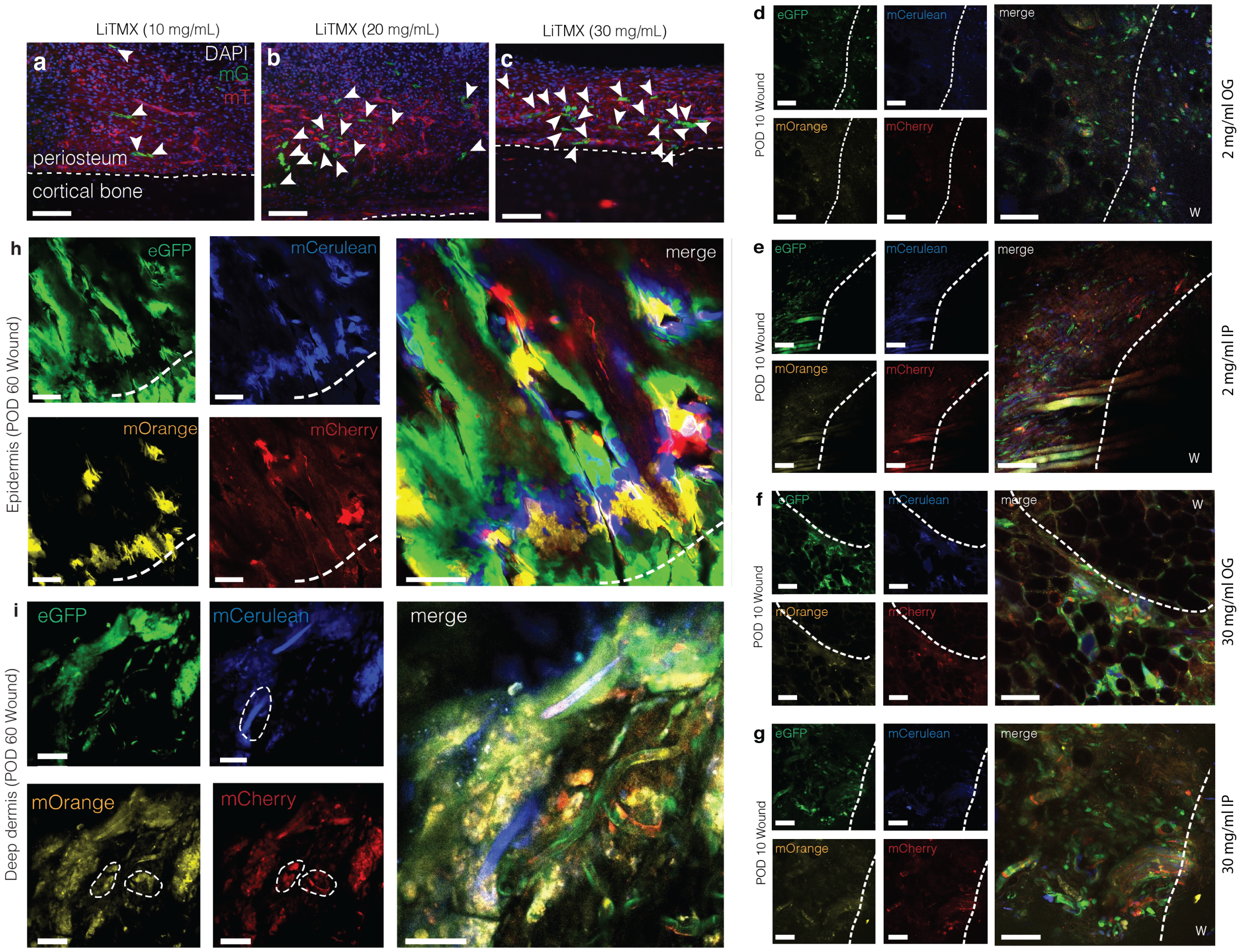

Supplementary Fig. 2. Dose-dependent labeling of the femoral periosteum and soft tissue fibroblasts

(**a–c**) Merged filters demonstrate the increasing frequency of labeled cells in the femoral periosteum with increased LiTMX dose in the *Gli1-CreER^{T2}::Rosa26-mTmG* mouse model (10 µg in (**a**), 20 µg in (**b**), and 30 µg in (**c**)) (white arrowheads highlight induced, GFP-labeled cells, white stains for DAPI, white dotted lines indicate the cortical bone – periosteum boundary).

(**d–g**) Standard techniques using systemic induction with tamoxifen in corn oil at a low dose (2mg/ml) via OG (**d**) or IP injection (**e**), or at a relatively high dose (30 mg/ml) via OG (**f**) or IP (**g**) shows patchy patterns of cell recombination and fluorescent labeling in complex tissue at POD 10 after cutaneous wounding, compared with local LiTMX application at comparable time points (**see Fig. 3 g–i**) (individual channels at left, merged at right, dotted white lines indicate wound edge, white "W"s indicate inner part of wound).

(h–i) Clonal expansion of epidermis and maintenance of wound-responsive vascular clones over time. (**h**) Whole mount confocal image of healed wound (white dotted line indicates wound

edge) at POD 60 shows expansion of epidermal keratinocytes involved in injury repair. (**i**) Whole mount confocal image of healed wound at POD 60 shows remodeling of dermal wound healing clones over time, with persistence of regions of vessel-associated cell clonality (white dotted outlines indicate individual colored clonal cell populations).

Experiments conducted with *n=3* biological replicates per timepoint (where applicable) per condition (unless otherwise indicated), 2 dorsal wounds per mouse, scale bars represent 200um (unless otherwise indicated).
